# Supplementary material for: Spontaneous crack healing in calcite reveals the influence of dynamic strain evolution and surface chemistry
Source: Nat Commun. 2026 Apr 1;17:4703. doi: 10.1038/s41467-026-71110-x (PMC13212580; doi:10.1038/s41467-026-71110-x)
Supplement: Supplementary file 1 — Supplementary Information [file 41467_2026_71110_MOESM1_ESM.pdf]

Supplementary Information

Spontaneous crack healing in calcite reveals the influence of dynamic strain evolution and surface chemistry

Michelle Devoe*[1]†, Harrison Lisabeth[2]†, Seiji Nakagawa[2], Zhao Hao[2], Nobumichi Tamura[3], Hans-Rudolf Wenk[1]

[1]Department of Earth and Planetary Science, University of California, Berkeley, 307 McCone Hall, Berkeley, CA 94720, USA

[2]Energy Geoscience Division, Lawrence Berkeley National Laboratory, 1 Cyclotron Road, Berkeley, CA 94720, USA

[3]Advanced Light Source, Lawrence Berkeley National Laboratory, 1 Cyclotron Road, Berkeley, CA 94720, USA

* Corresponding author: mdevoe@berkeley.edu

†: Authors contributed equally to this work

This PDF file includes:

Supplementary Figures 1 to 3
Supplementary Table 1

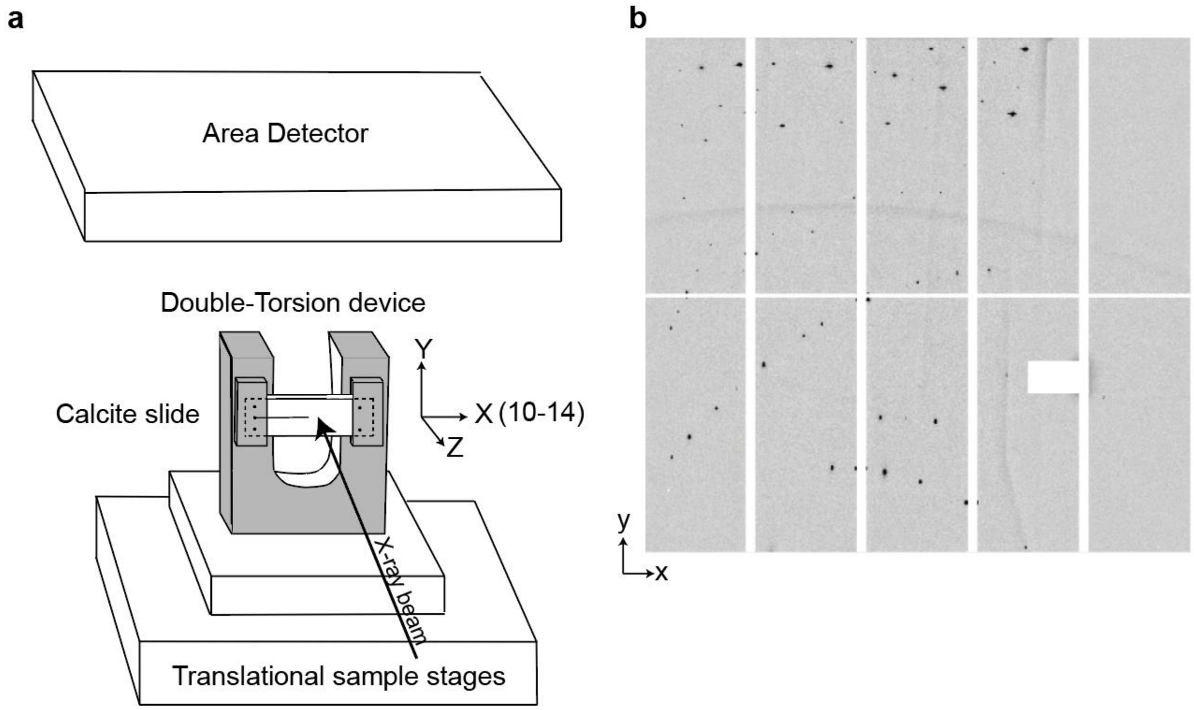

Supplementary Figure 1.

Experiment schematic. **(a)** Schematic of custom double-torsion device used to hold the calcite slide perpendicular to the X-ray beam. The device is located beneath the area detector in laboratory coordinate system (X, Y, Z) with the calcite cleavage plane (10-14) parallel to the X-axis. **(b)** Example diffraction image of calcite. Shadow from the double-torsion device is seen on the right-most panels. Diffraction pattern coordinate system of (x, y) is used.

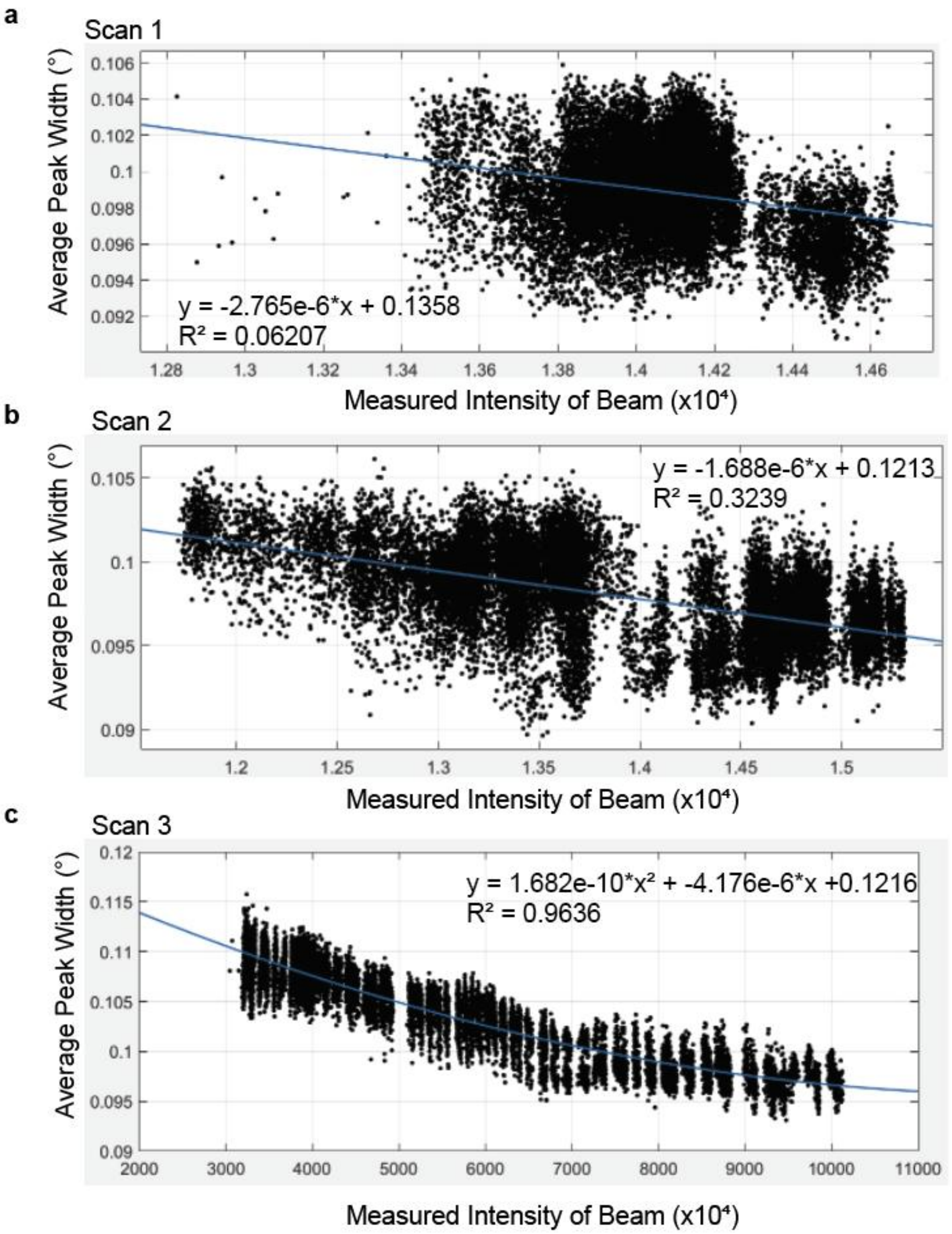

Supplementary Figure 2.

Peak width corrections. Measured X-ray beam intensity and average peak width plotted for **(a)** scan 1, **(b)** scan 2, **(c)** scan 3. Trendline fits are R^2 values used to correct peak width values for beam intensity are shown on each plot.

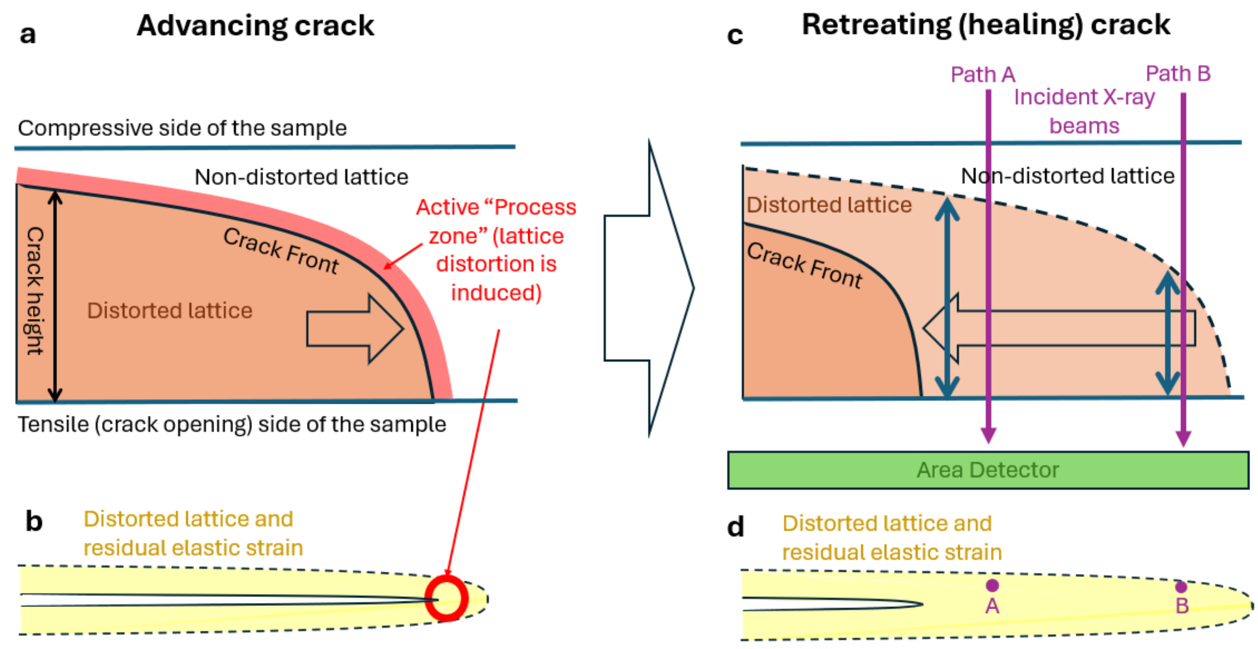

Supplementary Figure 3.

Cumulative lattice strain dependent on X-ray path relative to crack geometry. (a) Side profile of advancing mode I crack front through calcite sample in double torsion device, with compressive side of sample on top and tensile side of sample on the bottom. The "process zone" ahead of the crack front is marked in red to annotate the induction of plastic deformation/lattice distortion to the calcite lattice (orange shading). **(b)** Bird's eye view of the crack profile, showing the crack surrounded by a region of distorted lattice, affected by the procession of the crack front (yellow shading). **(c)** Side profile of retreating crack front, showing the distorted lattice profile following crack front recession, and two possible X-ray beam paths. Path A and Path B represent plausible X-ray beam paths that transverse different proportions of distorted lattice (double-headed blue arrows), and the area detector (green rectangle) would collect the averaged signal through the sample thickness. For ease of representation, the Area Detector which captures the diffraction pattern is positioned in X-ray transmission geometry. **(d)** Bird's eye view of the crack profile during crack retreat/healing, showing the crack surrounded by a region of distorted lattice, affected by the procession of the crack front (yellow shading) following load removal.

Supplementary Table 1. Minimum and maximum stress and strain values of calcite control scan.

	Von Mises stress (MPa)	ε_{xx} (10^{-3})	ε_{yy} (10^{-3})	ε_{zz} (10^{-3})
Min. value	366	1.69	0.734	-3.75
Max. value	512	2.52	1.59	-3.00
